# Single and combination treatment of *Toxoplasma gondii* infections with a bumped kinase inhibitor and artemisone *in vitro* and with artemiside in experimentally infected mice

**DOI:** 10.1016/j.exppara.2023.108655

**Published:** 2023-11-18

**Authors:** Carling Schlange, Joachim Müller, Dennis Imhof, Kai Pascal Alexander Hänggeli, Ghalia Boubaker, Luis-Miguel Ortega-Mora, Ho Ning Wong, Richard K. Haynes, Wesley C. Van Voorhis, Andrew Hemphill

**Affiliations:** a Institute of Parasitology, Department of Infectious Diseases and Pathobiology, Vetsuisse Faculty, University of Bern, Länggass-Strasse 122, CH-3012, Bern, Switzerland; b SALUVET, Animal Health Department, Faculty of Veterinary Sciences, Complutense University of Madrid, Ciudad Universitaria S/n, 28040, Madrid, Spain; c Rural Health Research Institute, Charles Sturt University, Orange, New South Wales, 2800, Australia; d Centre of Excellence for Pharmaceutical Sciences, Faculty of Health Sciences, North-West University, Potchefstroom, 2520, South Africa; e Center for Emerging and Re-emerging Infectious Diseases (CERID), Division of Allergy and Infectious Diseases, Department of Medicine, University of Washington School of Medicine, Seattle, WA, USA

**Keywords:** Apicomplexa, Chemotherapy, Model systems, Synergy

## Abstract

In previous studies, the artemisinin derivatives artemisone, its pro-drug artemiside and the bumped-kinase inhibitor BKI-1748 were effective against *T. gondii* via different modes of action. This suggests that they may act synergistically resulting in improved efficacies *in vitro* and *in vivo.* To test this hypothesis, the compounds were applied alone and in combination to *T. gondii* infected human fibroblast host cells in order to determine their inhibition constants and effects on cellular ultrastructure. In addition, the efficacy of either single- or combined treatments were assessed in an acute TgShSp1-oocyst infection model based on CD1 outbred mice. Whereas the IC_50_ of the compounds in combination (42 nM) was close to the IC_50_ of BKI-1748 alone (46 nM) and half of the IC_50_ of artemisone alone (92 nM), the IC_90_ of the combination was half of the values found with the single compounds (138 nM vs. ca. 270 nM). Another indication for synergistic effects *in vitro* were distinct alterations of the cellular ultrastructure of tachyzoites observed in combination, but not with the single compounds. These promising results could not be reproduced *in vivo*. There was no decrease in number of *T. gondii* positive brains by either treatment. However, the levels of infection in these brains, i. e. the number of tachyzoites, was significantly decreased upon BKI-1748 treatment alone, and the combination with artemiside did not produce any further decrease. The treatment with artemiside alone had no significant effects. A vertical transmission model could not be established since artemiside strongly interfered with pregnancy and caused abortion. These results show that is difficult to extrapolate from promising *in vitro* results to the situation *in vivo*.

## 1. Introduction

*Toxoplasma gondii* is an apicomplexan parasite that causes toxoplasmosis in animals and humans, a disease of high medical, veterinary and economic importance worldwide (Daher et al., 2021). *T. gondii* infections have been reported from most mammalian and several bird species, and approximately 25% of the human population is chronically infected with this parasite. However, only a fraction actually develops acute and thus clinical disease (Dunay et al., 2018).

Throughout its life cycle, *T. gondii* undergoes three distinct infectious stages: (i) a rapidly dividing tachyzoite stage that undergoes a lytic cycle characterized by invasion, intracellular proliferation within a parasitophorous vacuole, egress and infection of neighboring cells. Tachyzoites cause acute toxoplasmosis; (ii) a slowly dividing bradyzoite stage, which forms intracellular tissue cysts surrounded by a cyst wall that protects these parasites from immunological and physiological reactions on part of the hosts, and represents the chronic stage; (iii) the oocyst stage of high tenacity, which is the end-product of a sexual process taking place in the intestinal tissue of the feline definitive host, and which is released into the environment by fecal shedding and undergoes sporulation to form infective sporozoites (Robert-Gangneux and Dardé, 2012). Bradyzoites and sporozoites are orally infective, and differentiate into tachyzoites which disseminate throughout the host. In immune-competent hosts, these tachyzoites encounter a severe immunological response that initiates differentiation into bradyzoites and cyst formation. Thus, infection normally develops asymptomatically or with only mild and transient symptoms. In immune-compromised patients, however, life-threatening effects can occur, as tachyzoites do not undergo bradyzoite differentiation, and thus cause severe acute toxoplasmosis, either through recently acquired infection, or through reactivation of bradyzoites that re-differentiate into rapidly proliferating tachyzoites when immunity is impaired. An additional route of transmission is congenital infection of the placental tissue and the fetus by tachyzoites, that occurs upon primary infection of the mother during pregnancy, possibly leading to abortion, malformations, or neurological symptoms in the fetus such as hydrocephalus, often with lethal outcome. Other events include ocular toxoplasmosis, or long-term effects affecting brain functions (Attias et al., 2020; Daher et al., 2021). Besides being a significant medical problem, *T. gondii* causes severe diseases in pets and in small ruminants and many other food animal species, thus inflicting significant losses in livestock farming (Lindsay and Dubey, 2020). Even though the burden of toxoplasmosis is one of the highest on the list of all parasitic diseases, there is currently no efficient vaccine on the market, with the exception of a live-attenuated strain (*T.gondii* S48) that is marketed for sheep only (Wang et al., 2019). Existing drugs used for treatment typically include antifolates such as a combination of pyrimethamine-sulfadiazine or trimethoprim-sulfamethoxazole, and pyrimethamine combined with clindamycin, azithromycin, or atovaquone. These drugs can cause serious adverse effects and are not always efficacious (Dunay et al., 2018; Djurković-Djaković et al., 2019). In addition, currently marketed treatments are only effective against tachyzoites in the acute phase of infection (Alday and Doggett, 2017).

A promising class of compounds that are being developed for the treatment of toxoplasmosis are bumped kinase inhibitors (BKIs), a class of ATP-competitive kinase inhibitors designed to inhibit the activity of calcium-dependent protein kinase 1 (CDPK1) in a variety of apicomplexan parasite species. CDPK1 is crucially involved in host cell invasion and represents an excellent drug target, since this kinase does not exist in mammals (Billker et al., 2009; Choi et al., 2020). BKIs have been designed to fit into a hydrophobic sub-pocket of the CDPK1 ATP binding pocket, which has glycine as a small gatekeeper residue. In contrast, the bulkier gatekeeper residues in the ATP-binding pocket of mammalian kinases impair binding of BKIs, thus minimizing inhibition of kinases in the mammalian host (Van Voorhis et al., 2017). The *in vitro* and *in vivo* action of different BKIs have been investigated and several derivatives demonstrated auspicious efficacy against *T. gondii* and related apicomplexans (Choi et al., 2020; Van Voorhis et al., 2021), including the 5-aminopyrazole-4-carboxamide BKI-1748 (Imhof et al., 2021). BKI-1748 was shown to be safe and efficacious with IC_50_-values of 43 nM against *T. gondii in vitro*, and exhibited favorable pharmacokinetic properties after oral application (Huang et al., 2019; Imhof et al., 2021). In addition, treatment with BKI-1748 led to clear inhibition of transplacental transmission, increased pup survival and decreased cerebral infection in a pregnant toxoplasmosis mouse model based on oocyst infection (Imhof et al., 2021). BKI-treatments of *T. gondii,* and of the closely related *Neospora caninum*, demonstrated that these drugs affect host cell invasion and egress but do not act parasiticidal. Instead, they induce the formation of multinucleated complexes (MNCs) (Imhof et al., 2021; Winzer et al., 2015, 2020a, 2020b). These MNCs are schizont-like complexes composed of newly-formed zoites that are blocked in the final stage of cytokinesis. These zoites are surrounded by the inner membrane complex but lack the outer tachyzoite plasma membrane and remain intracellular and viable for extended periods of time (Imhof et al., 2021). It is anticipated that the parasite converts into the MNC-stage to evade the drug pressure and to ensure its survival. In line with this, comparative proteomics of MNCs and tachyzoites of the closely related *N. caninum* showed that MNCs exhibit several upregulated bradyzoite markers (Winzer et al., 2020b), and that MNC formation was a reversible process, with tachyzoites re-emerging out of the complexes and resuming proliferation after drug-discontinuation (Winzer et al., 2020a).

Artemisinin derived from the plant *Artemisia annua* and its derivatives (ARTs), originally derived from the plant *Artemisia annua*, are used as a first-line therapy against malaria, caused by *Plasmodium* sp. Apart from their antimalarial activity, ARTs have been repurposed as potential drugs against diseases caused by other protozoan parasites including *Leishmania* spp.*, Trypanosoma* spp.*, Toxoplasma gondii, Neospora caninum, Eimeria tenella, Acanthamoeba castellanii, Naegleria fowleri, Cryptosporidium parvum, Giardia lamblia,* and *Babesia* spp. (Loo et al., 2017). Two of these derivatives, namely the amino-artemisinins artemisone (GC003) and artemiside (GC008), exhibited promising activity when tested *in vitro* against *T. gondii* with IC_50_ values of 50–70 nM (Müller et al., 2023). Upon oral application of artemiside, this compound is rapidly metabolized to the sulfoxide artemisox and then into artemisone (Gibhard et al., 2021; Watson et al., 2021). Artemisone and artemiside have been previously assessed for efficacy against *T. gondii* infection, either by subcutaneous application in outbred CD1 mice infected with a sublethal dose of *T. gondii* tachyzoites, or in inbred C57BL/6 female gamma interferon knock-out (IFN-γ ko) mice orally infected with *T. gondii* tissue cysts. Results were promising in that these two compounds were able to control the infection, but they did not result in a complete cure (Dunay et al., 2009).

In contrast to BKIs that affect invasion and egress and induce MNC-formation, amino-artemisinins such as artemisone induce distinct alterations in the parasite mitochondrion, including the loss of the matrix and cristae, and swelling of the mitochondrial lumen (Müller et al., 2023). Thus, they obviously exhibit differences in their mechanisms of action. Based on these results, we formulate the hypothesis that a combined treatment with both artemisone and BKI-1748 results in synergistic effects. To test this hypothesis, we quantified the *in vitro* efficacies of both compounds either individually or as a combined treatment against *T. gondii* tachyzoites. Moreover, we investigate the impact of individual and combined treatments on the parasite morphology and ultrastructure. To verify whether the observed *in vitro* effects coud be reproduced *in vivo*, artemiside, the prodrug of artemisone, BKI-1748, and an artemiside/BKI-1748 combination treatment were assessed in a toxoplasmosis mouse model based on oocyst infection.

## 2. Materials and methods

### 2.1. Chemicals and compound preparation

If not otherwise stated, all biochemical reagents were from Sigma (St. Louis, MO, USA). Culture media were purchased from Thermo Fisher Scientific (Waltham, MA, USA). Artemisone (GC003) and Artemiside (GC008), each >95% pure were prepared and purified as previously described (Gibhard et al., 2021; Coertzen et al., 2018). BKI-1748 was originally synthesized in the Department of Biochemistry of the University of Washington, USA and scaled up by WuXi Apptec Inc., Wuhan, China to >98% purity by LC/MS-MS and NMR and shipped as powder. For *in vitro* studies, stock solutions of 20 mM were prepared in dimethyl sulfoxide (DMSO) and stored at − 20 °C. For *in vivo* experiments, compounds were suspended in sterile corn oil prior to administration of mice by oral gavage.

### 2.2. Host cell and parasite culture

Human foreskin fibroblasts (HFF) were maintained in Dulbecco’s Minimal Essential Medium (DMEM) containing 10 % fetal calf serum (FCS) at 37 °C/5% CO_2_. *T. gondii* ME49 and *T. gondii* RH strain constitutively expressing β-galactosidase (*T. gondii* β-gal) were maintained by serial passages in HFF. *T. gondii* oocysts of the type II isolate TgShSp1 (Sánchez-Sánchez et al., 2019) were obtained from Complutense University of Madrid, Spain, and stored at 4 °C until use.

### 2.3. In vitro efficacy assessment of artemisone and BKI-1748 individually or in combination against T. gondii

Drug efficacy tests of artemiside, BKI-1748 and a combination of both drugs were performed using a transgenic *T. gondii* β-gal strain that constitutively expresses Beta-Galactosidase (McFadden et al., 1997) using the methodology described earlier (Anghel et al., 2021; Müller et al., 2023). Confluent HFF monolayers were cultured in 96-well-plates and infected with 10^3^ *T. gondii* β-gal tachyzoites. Serial dilutions of artemiside, BKI-1748 and the combination were prepared in cell culture medium (0–1 μM) and added 5 min before infecting the host cells. After 72 h of culture at 37 °C/5% CO_2_, the medium was aspirated, and cells were permeabilized by adding 90 μl PBS containing 0.05% Triton X-100. After the addition of 10 μl 5 mM chlorophenol red-β-Dgalactopyranoside (CPRG; Roche Diagnostics, Rotkreuz, Switzerland) dissolved in PBS, the absorption shift was measured at 570 nm wavelength at various time points using an EnSpire^®^ multimode plate reader (PerkinElmer, Inc., Waltham, MA, USA). The activity measured as the release of chlorophenol red over time was proportional to the number of live parasites down to 50 per well as determined in pilot assays. IC_50_ values were calculated after the logit-log-transformation of relative growth and subsequent regression analysis.

### 2.4. Immunofluorescence labelling of T. gondii tachyzoites treated with artemisone and BKI-1748 individually or in combination

Immunofluorescence staining was performed as described previously (Anghel et al., 2021). Briefly, 3 × 10^4^ HFF were grown on glass coverslips and infected 1 day after with 3 × 10^4^ *T. gondii* ME49 tachyzoites. 4 h after infection at 37 °C/5% CO_2_, the medium was replaced by medium supplemented with 1 μM artemisone, 1 μM BKI-1748, 1 μM artemisone +1 μM BKI-1748 or by medium with 0.01% DMSO as control. The cultures were maintained further and every third day medium with or without drug was exchanged by freshly prepared one. Infected cells were fixed on day 1, 3, 6, and 9 in 3% paraformaldehyde in PBS (pH 7.2) for 10 min, and permeabilized in a 1:1 solution of precooled methanol/acetone at (− 20 °C) for 15 min. After rehydration, coverslips were kept in PBS/3% bovine serum albumin (BSA) overnight at 4 °C to block nonspecific binding sites. For labelling, the monoclonal mouse anti-SAG1 antibody (diluted 1:1000) directed against the *T. gondii* surface antigen 1, anti-mouse fluorescein-isothiocyanate (FITC) (diluted 1:300), polyclonal rabbit anti-IMC1 (diluted 1:500) directed against the inner membrane complex, and anti-rabbit tetramethyl-rhodamine-isothiocyanate (TRITC) (diluted 1:300) were applied one after the other. Following, coverslips were mounted in H-1200 Vectashield mounting medium (Vector Laboratories, Bulingame, CA, USA) containing 4,6-diamidino-2-phenylindole (DAPI) for nuclei staining. All specimens were viewed on a Nikon Eclipse E800 digital confocal fluorescence microscope.

### 2.5. Transmission electron microscopy (TEM)

Transmission-electron microscopy (TEM) preparation was performed as previously described (Anghel et al., 2021; Müller et al., 2023). In short, 5 × 10^5^ HFF were grown in T25 tissue culture flasks and infected after 4 days after with 2.5 × 10^6^ *T. gondii* ME49 tachyzoites. 4 h after infection, medium was replaced by medium supplemented with 1 μM artemisone, 1 μM BKI-1748, 1 μM artemisone + BKI-1748 or 0.01% DMSO for solvent control, and flasks were incubated at 37 °C/5% CO_2_. After 3 days of culture, medium was exchanged with freshly prepared one plus/minus corresponding drug. Fixation of samples was carried out after 6, 24, 48, 72 and 96 h after start of treatment by washing flasks twice with 0.1 M Na-Cacodylate buffer (pH 7.3) before addition of 2% glutaraldehyde in 0.1 M Na-Cacodylate buffer for 10 min at room temperature. Subsequently, infected monolayers were removed from the flasks using a cell scraper, and fixation was continued further overnight at 4 °C. The specimens were washed three times in cacodylate buffer and post-fixed in 2% osmium tetroxide in cacodylate buffer. Following three washes in water, samples were pre-stained in UranyLess (Electron Microscopy Science, Hatfield, PA, USA) solution for 30 min and afterwards dehydrated in graded series of ethanol (30%, 50%, 70%, 90% and 3 × 100%). For embedding, samples were immersed in Epon-812 epoxy resin with three exchanges of resin every 4–8 h. Polymerization at 60 °C was done overnight before cutting ultrathin sections using an ultramicrotome (Reichert & Jung, Vienna, Austria). Sections were placed onto 300 mesh formvar/carbon coated nickel grids (Plano GmbH, Wetzlar, Germany), and specimens were viewed on a Philips CM12 TEM operating at 80 kV.

### 2.6. Ethic statement

Experiments involving animals were approved by the Animal Welfare Committee of the Canton of Berne (license 117/20). Animals were handled in strict accordance with the practices to minimize suffering. Female CD1 mice were purchased at 6 weeks of age from Charles River (Sulzberg, Germany) and maintained in a common room under controlled temperature and a 14 h/10 h light and dark cycle, with food and water accessible *ad libitum*. For acclimatization, the animals were housed in the facility 2 weeks prior to experimentation and procedures were carried out according to the guidelines of the animal welfare legislation of the Swiss Veterinary Office.

### 2.7. Pregnancy interference test in BALB/c mice

Potential pregnancy interference of artemiside treatment in mice was evaluated as described earlier (Anghel et al., 2020). 12 female BALB/c mice were estrus-synchronized for 3 days and 1 male and 2 females per cage were housed together for 72 h. After mating, males were removed, and female mice were randomly allocated to 2 experimental groups (6 mice/group; 2 females/cage). Artemiside formulated in corn oil was supplied by oral gavage of 100 μl at 20 mg/kg/day on day 9–13 post-mating, while the placebo group received corn oil only. Mice were observed daily, and weight measurements were carried out every third day. At day 18 post-mating, pregnant females were separated into single cages to rear their offspring. Pregnant mice gave birth on days 20–22. Data on fertility, litter size, neonatal mortality, postnatal mortality and the clinical state of dams and pups were recorded daily. Dams and pups were observed for at least 2 weeks, before they were euthanized in a chamber by isoflurane/CO_2_.

### 2.8. Evaluation of artemiside and BKI-1748 treatment-effects as single compounds or in combination in a non-pregnant toxoplasmosis mouse model

32 female 8-week-old CD1 mice were randomly distributed to 4 experimental groups (8 mice/group; 4 mice/cage): artemiside; BKI-1748; artemiside + BKI-1748; C+ (corn oil); before the infection, blood samples of 2 mice per group were collected. All mice were infected with 400 *T. gondii* ShSp1 oocysts suspended in 100 μl PBS, applied by oral gavage. 2 days post-infection (p.i.), treatment was initiated. For this, compounds were immersed in corn oil and administered by oral gavage at 20 mg/kg/day in 100 μl for 5 consecutive days, whereby the positive control group received corn oil only. During the following 3 weeks, mice were checked regularly for clinical signs. 28 days p. i., all mice were euthanized by isoflurane/CO_2_. Blood, brain, and eye samples were collected for further analysis. Quantification of the parasite load was performed by real-time PCR methods designed for *T. gondii* (Costa et al., 2000). The NucleoSpin DNA RapidLyse Kit (Macherey-Nagel, Oensingen, Switzerland) was applied for DNA purification and DNA concentrations were quantified with the QuantiFluor double-stranded DNA (dsDNA) system (Promega, Madison, WI, USA). Quantitative real-time PCR was performed with the Light Cycler (Roche, Basel, Switzerland), and parasite loads were calculated with a standard curve of DNA samples from 1′000, 100, 10 *T. gondii* tachyzoites included in each run. To assess the antibody response of the mice against *T. gondii*, the blood serum level of immunoglobulin G (IgG) directed against *T. gondii* RH protein extract was measured by enzyme-linked immunosorbent assay (ELISA) as described earlier (Sánchez-Sánchez et al., 2019).

### 2.9. Statistical analysis

The concentrations that exert 50% inhibition of tachyzoite proliferation (IC_50_) were calculated using the logit-log algorithm as described (Müller and Hemphill, 2013). The concentrations that exert 90% inhibition of tachyzoite proliferation (IC_90_) was calculated using the Hill factor H, the absolute value of the slopes of the logit-log via the formula IC_90_=IC_50_ × 9^1/H^ (Tjorve and Tjorve, 2017). Nominal data were compared by chi-square tests for independence. Parasite burdens, and antibody titers were compared between groups by the non-parametric Kruskal–Wallis test, followed by a Mann–Whitney-U test. Statistical analysis was performed using Graphpad Prism version 9.3.1 for MacOS (GraphPad Software, La Jolla, CA, USA, www.graphpad.com).

## 3. Results

### 3.1. In vitro efficacies of artemisone and BKI-1748, applied as single drugs or in combination

**Fig. 1.**
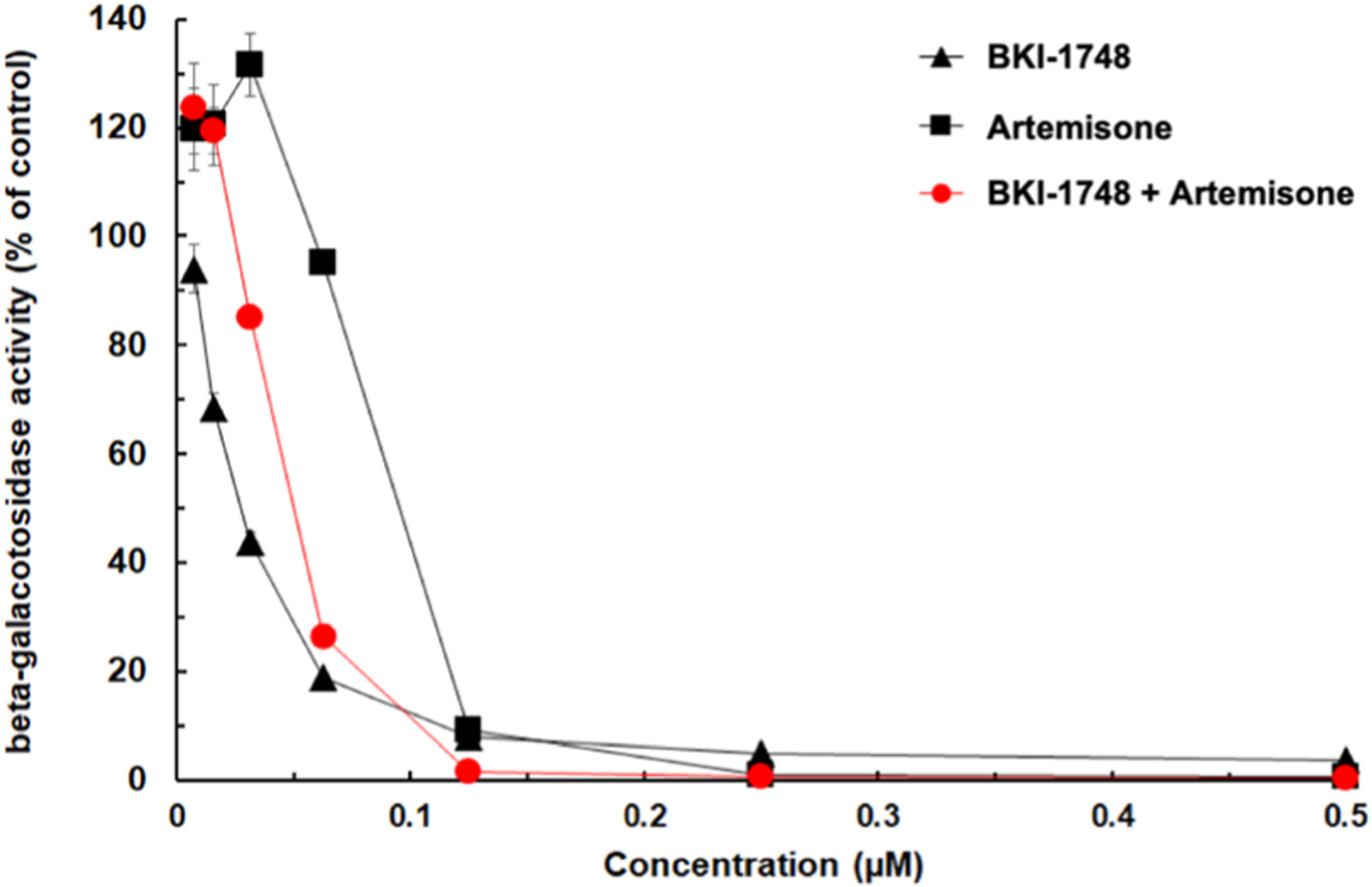
Dose-response curve of transgenic *T. gondii* RH tachyzoites constitutively expressing beta-galactosidase grown in HFF and treated with different concentrations (0–0.5 μM) of BKI-1748, artemisone and equimolar concentrations of both compounds.

The outcomes of dose-responses of BKI-1748, artemisone and equimolar combinations of both compounds against tachyzoites of a *T. gondii* RH β-gal reporter strain are shown in Fig. 1 and Table 1. Upon addition of compounds concomitantly to the infection of HFF monolayers, IC_50_ values of 46 nM for BKI-1748 and 92 nM for artemisone demonstrated a potent activity when applied alone. When the two compounds were combined, 50% inhibition of parasite proliferation was seen at 42 nM. However, the IC_90_ of artemisone and BKI-1748 was basically identical (269 nM for BKI-1748 and 271 nM for artemisone), whereas with the combination of both compounds 138 nM of each was sufficient to inhibit *T. gondii* tachyzoite proliferation by 90 %. At concentrations of 250 nM and higher of either artemisone or the artemisone/BKI-1748, the β-gal activity was below the detection limit.

### 3.2. Combined BKI-1748-artemisone treatment in vitro results in diminished growth of multinucleated complexes compared to treatment with BKI-1748 alone

**Fig. 2.**
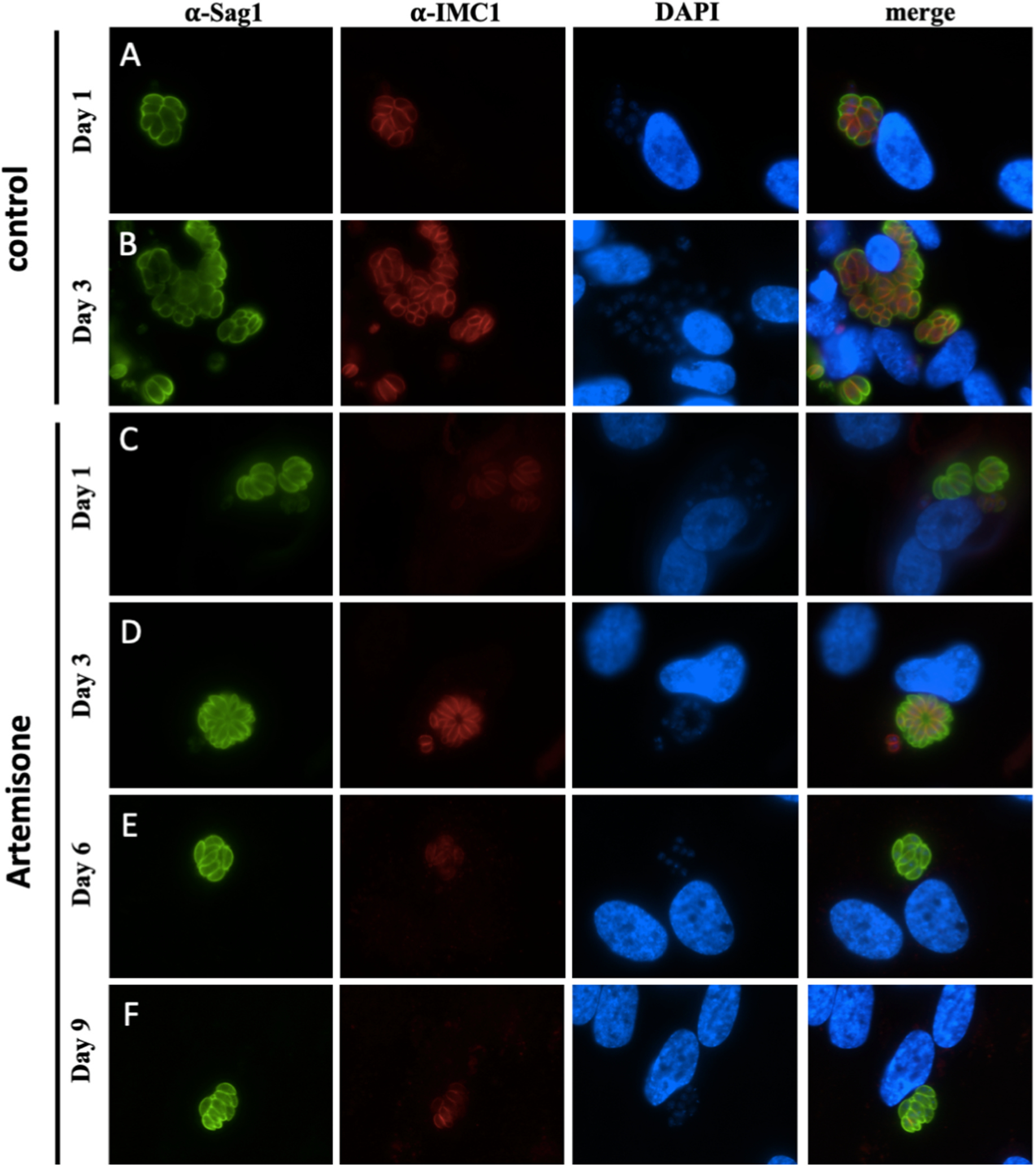
Immunofluorescence staining of *T. gondii* ME49 tachyzoites cultured in HFF either in the presence of a solvent control (A, B) or in the presence of artemisone (C–F) for different timespans as indicated. TgSAG1 is indicated in green, IMC1 labeling is shown in red.

**Fig. 3.**
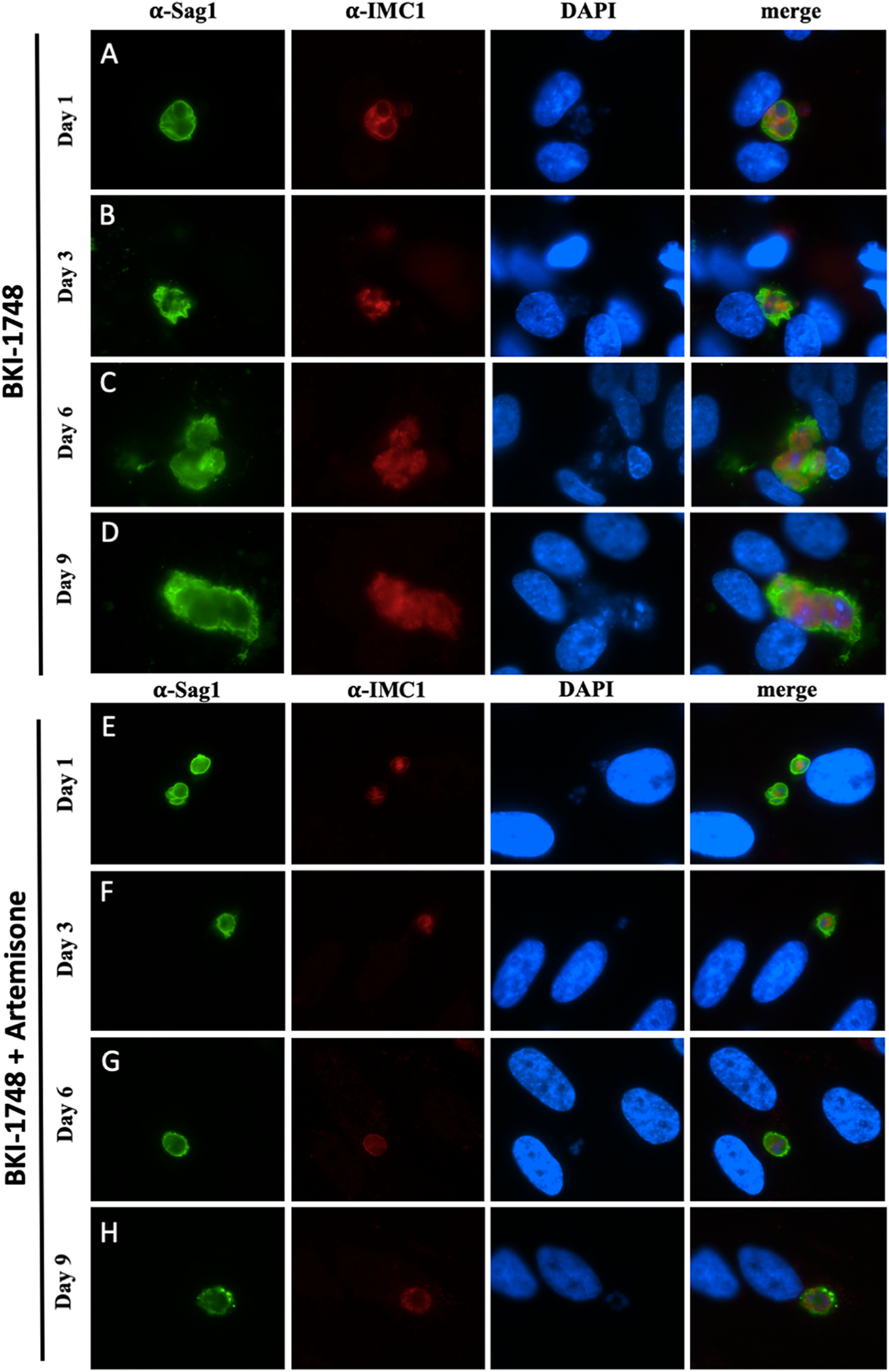
Immunofluorescence staining of *T. gondii* ME49 tachyzoites cultured in HFF either in the presence of BKI-1748 alone (A–D) or of BKI-1748 combined to artemisone (E–H). TgSAG1 is indicated in green, IMC1 labeling is shown in red.

*T. gondii* tachyzoite *in vitro* proliferation and growth of parasitophorous vacuoles were visualized in infected cultures, with treatments starting 4 h after infection, by immunofluorescence using antibodies directed against TgSAG1, the major tachyzoite surface antigen, and TgIMC1, which serves as a zoite proliferation marker (Fig. 2A and B). Tachyzoites treated with artemisone (Fig. 2C–F) were still undergoing endodyogeny and formed rosettes and parasitophorous vacuoles. However, compared to the non-treated control, proliferation was markedly inhibited. Although IMC1 was expressed by all tachyzoites, staining intensity was diminished at day 1 and later at day 6 and 9 of treatment. In contrast, treatments of *T. gondii* tachyzoites with BKI-1748 (Fig. 3A–D) did not lead to classical rosettes, but the formation of multinucleated complexes (MNCs), which exhibited TgSAG1 surface labeling and interior IMC-staining as previously shown (Imhof et al., 2021). Treatments of *T. gondii* cultures with the combination of artemisone and BKI-1748 resulted in the formation of smaller parasitophorous vacuoles containing only few nuclei, which did not notably increase in size during the 9-day treatment period, and also exhibited TgSAG1 labeling as well as IMC1-labeling, although at low intensity (Figure E–H).

### 3.3. Ultrastructural effects of artemisone, BKI-1748 and combined artemisone-bki-1748 treatment

**Fig. 4.**
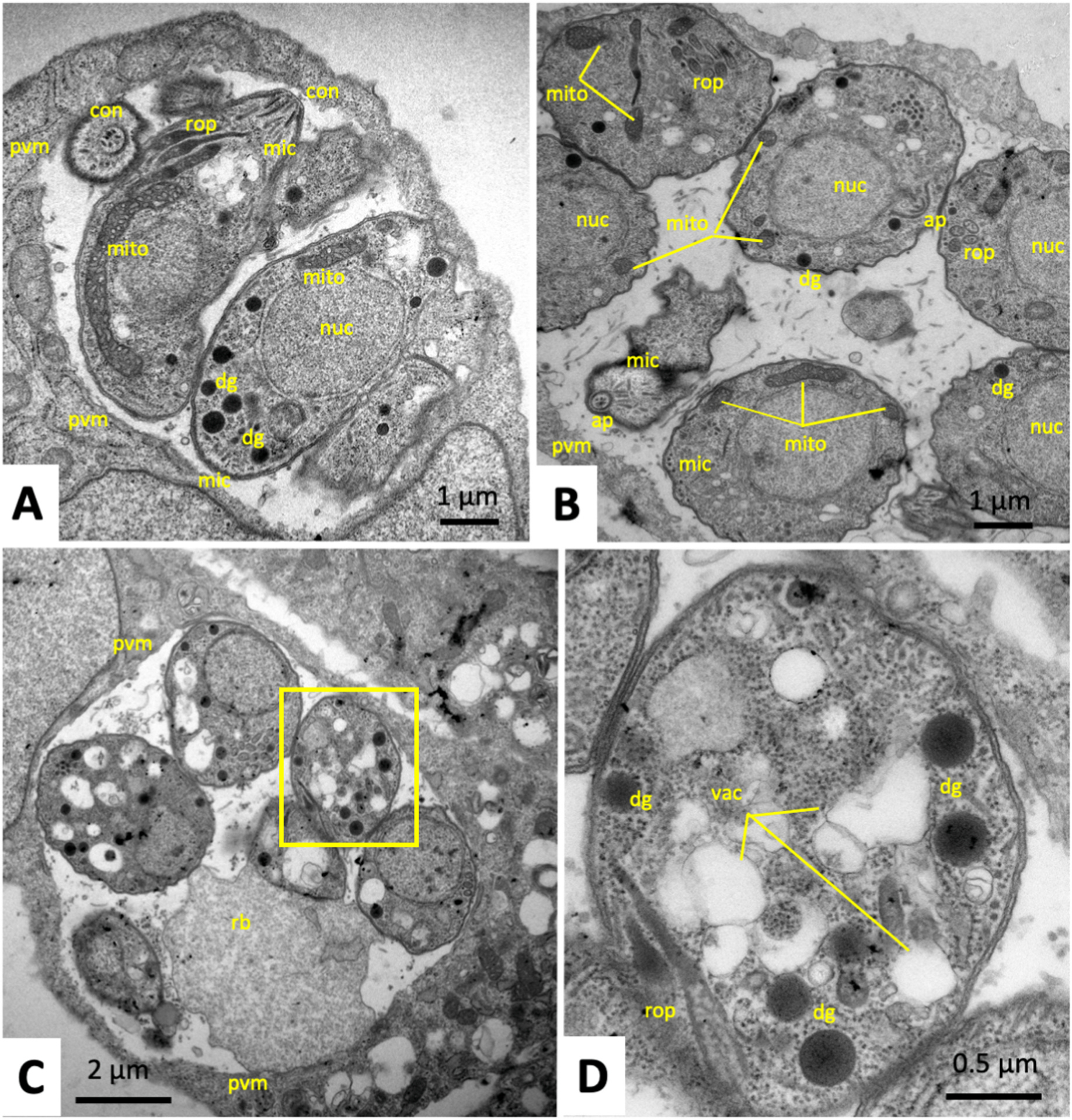
Transmission electron microscopy of *T. gondii* ME49 tachyzoites grown in HFF. A and B are examples of non-treated tachyzoites proliferating for 24 and 48 h, respectively). Typical features such as micronemes (mic), rhoptries (rop), dense granules (dg), and the conoid (con) are clearly discernible. C and D show tachyzoites treataed with artemiside for 72 h. The boxed area in C is enlarged in D. pvm = parasitophorous vacuole membrane, nuc = nucleus, mito = mitochondrion, vac = vacuoles, rb = residual body.

As could be seen by TEM, in untreated control specimens (treated only with medium containing the DMSO solvent) fixed and processed after 24 h (Fig. 4 A) and 48 h p. i. (Fig. 4 B), intracellular tachyzoites had formed a parasitophorous vacuole surrounded by a parasitophorous vacuole membrane (PVM). The typical structural features of tachyzoites such as the apical portion with the conoid, secretory organelles including rhoptries, micronemes and dense granules, and the mitochondrion, characterized by an electron-dense matrix, were clearly discernible. Tachyzoites contain only one mitochondrion composed of branched tubules, thus only small portions of the entire mitochondrion are visible in one section plane.

**Fig. 5.**
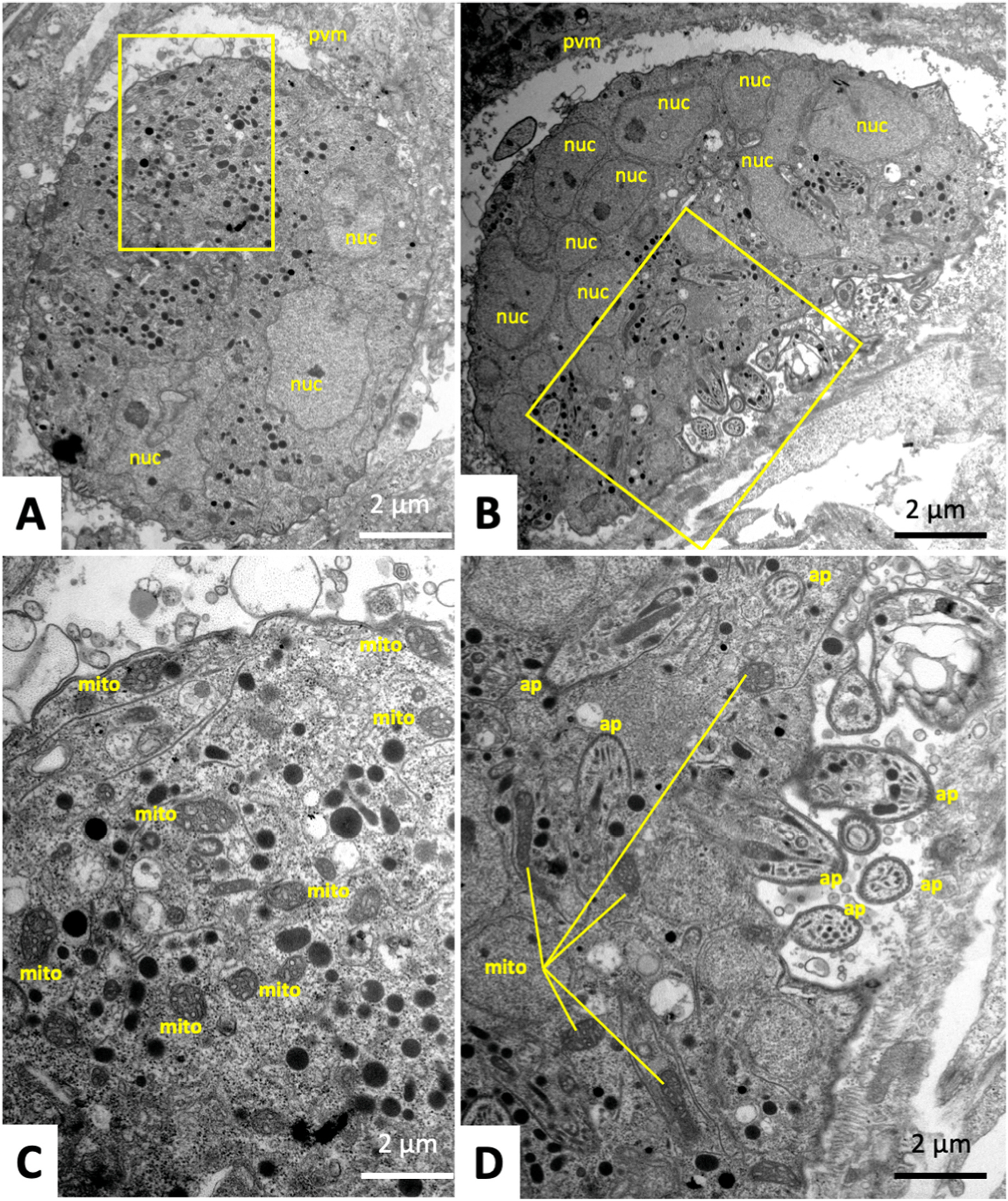
Transmission electron microscopy of *T. gondii* ME49 tachyzoites grown in HFF and treated with BKI-1748 for 48 (A) and 72 h (B). The boxed areas in a and B are shown as higher magnification views in C and D, respectively; nuc = nucleus, pvm = parasitophorous vacuole membrane, ap = apical portions of emerging zoites, mito = mitochondrion.

**Fig. 6.**
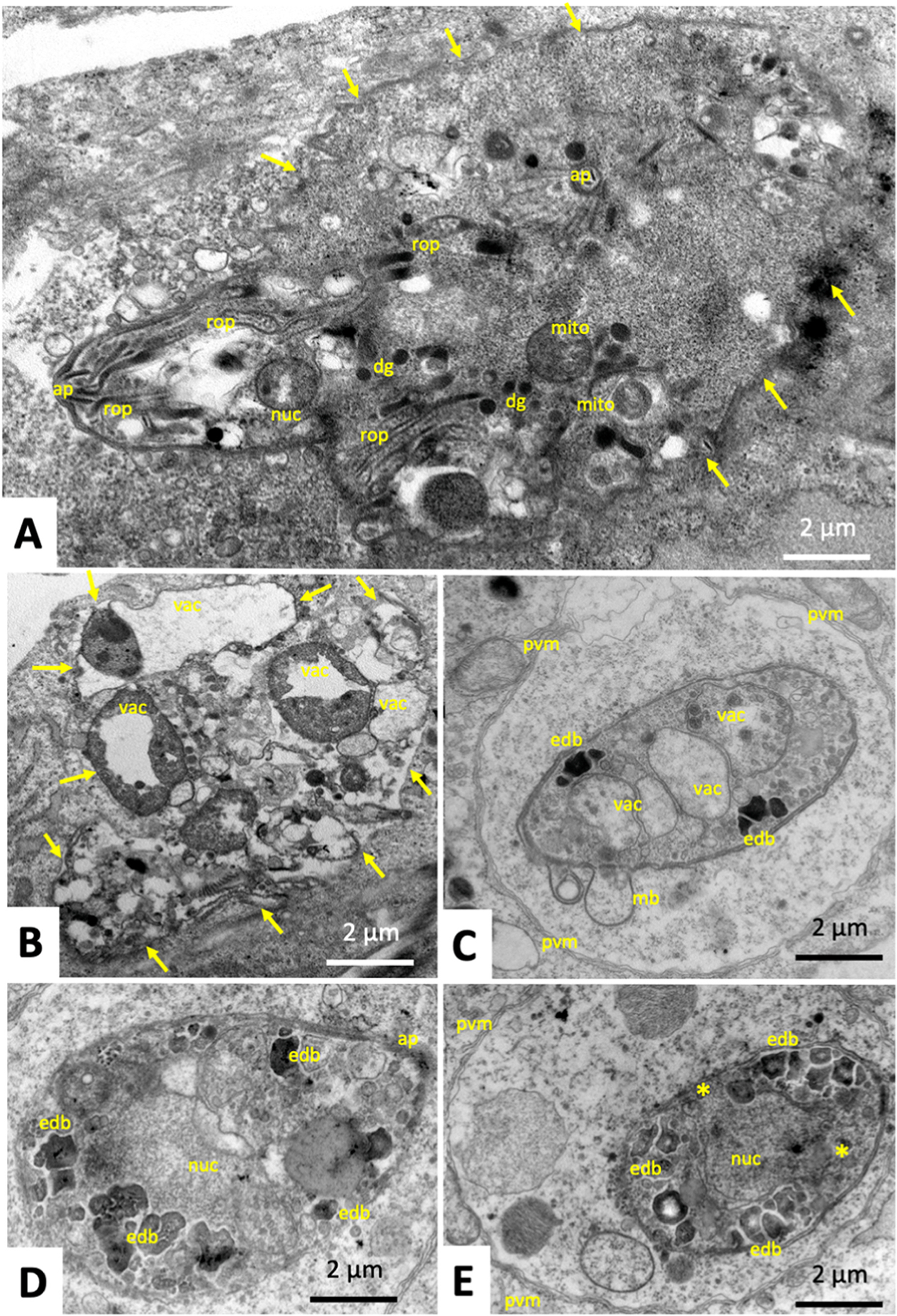
Transmission electron microscopy of *T. gondii* ME49 tachyzoites grown in HFF and treated with BKI-1748 and artemisone during 48 h (A), 72 h (B–D) or 96 h (E); rop = rhoptries, dg = dense granules, nuc = nucleus, ap = apical portion of emerging zoites, pvm = parasitophorous vacuole membrane, mito = mitochondrion, vac = vacuole, edb = electron dense body. Arrows point towards the pvm of multinucleated complexes.

Tachyzoites in cultures treated with artemisone during 48–72 h (Fig. 4 C, D) were largely lacking clearly discernible mitochondria, but instead the cytoplasm was heavily vacuolized. The parasitophorous vacuole and tachyzoite plasma membrane, however, remained intact. In contrast, treatments of *T. gondii* tachyzoites with BKI-1748 triggered the formation of schizont-like MNCs composed of several nuclei and newly formed zoites (Fig. 5A and B), which displayed a structurally intact mitochondrial matrix and cristae (Fig. 5 C), as well as micronemes, rhoptries and dense granules similar to untreated parasites. These MNCs exhibited a largely polarized organization, with nuclei concentrated close together and the newly formed apical parts of zoites emerging from the MNC periphery at the opposite end, as shown in Fig. 5 D. Treatment of *T. gondii* ME49 with the combination of both drugs, artemisone and BKI-1748 produced different phenotypes (Fig. 6). On one hand, MNC-like complexes with emerging apical parts of newly formed zoites could be detected and were visible during the first 48 h of treatment (Fig. 6 A). After later timepoints, however, only largely deteriorated and vacuolized residues of MNCs were seen, which had lost ultrastructural features typical of apicomplexans as exemplified after 72 h in Fig. 6 B. In addition, TEM detected many parasitophorous vacuoles that apparently contained only single parasites (Fig. 6C–E), but which were structurally altered, either through cytoplasmic vacuolization, the formation of cytoplasmic inclusion bodies of high electron density and/or the presence of surface membrane blebs (Fig. 6 E). In addition, the corresponding parasitophorous vacuole matrix was frequently filled with granular and electron-dense material of unknown composition (Fig. 6 E). Overall, the combined artemisone/BKI-1748 treatment exerted more pronounced ultrastructural damage than the individual treatments with each drug.

### 3.4. Efficacy of artemiside and BKI-1748, applied as single drugs or in combination in CD1 mice infected with T. gondii ShSp1 oocysts

In a primary pilot experiment, artemiside, which is rapidly metabolized to artemisone (Gibhard et al., 2021), was assessed in pregnancy interference assays using non-infected pregnant BALB/c mice (Table 2). In the artemiside treatment group, treated by oral application of artemiside (suspended in corn oil at 10 mg/kg/day from day 7–11 of pregnancy), none of the mice were found to be pregnant, and no offspring were born. In contrast, four out of the six mice in the group treated with corn oil became pregnant, overall 23 offspring mice were born, of which one succumbed to neonatal mortality on day 2 post-partum, and 22 developed normally until day 14 after birth. Thus, while there was a clear interference in pregnancy by artemiside treatment, the adult mice did not show any clinical signs or variations in weight. Thus, efficacy studies were carried out only in adult mice.

**Fig. 7.**
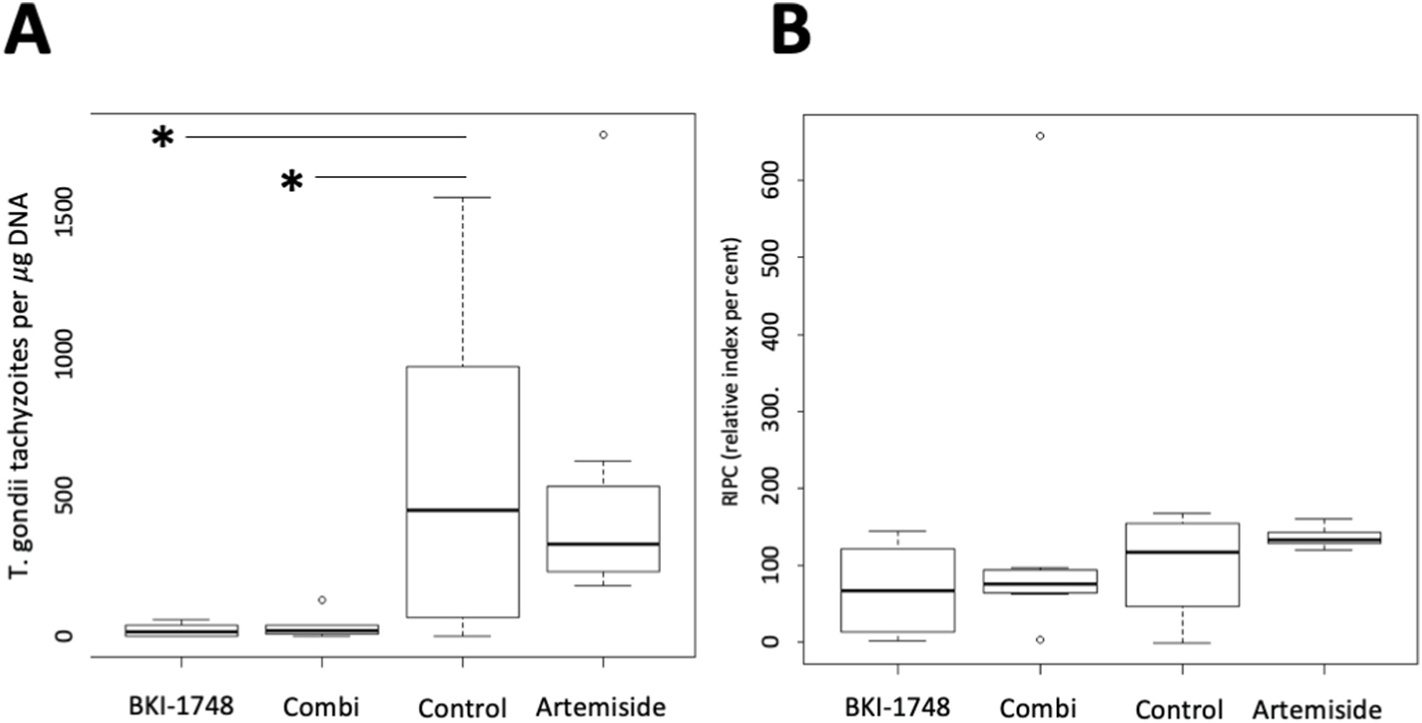
Outcome of peroral *in vivo* treatment of *T. gondii* ShSp1 oocyst infected CD1 mice with BKI-1748, artemisone, BKI-1748 + artemisone applied orally at 2 days post-infection. The control group was treated with corn oil only. Mice were euthanized and samples were collected at 4 weeks post-infection. The cerebral parasite load as determined by real time PCR and expressed as number of tachyzoites per μg DNA is shown in A. IgG responses in each experimental group are shown in B, expressed as the mean of RIPC (relative index per cent) compared to the respective positive control.; * indicates statistically significant differences in cerebral parasite load (non-parametric Kruskal–Wallis test, followed by a Mann–Whitney-U test).

Upon infection of CD1 mice with *T. gondii* ShSp1 oocysts, all mice were observed twice daily and weighed weekly. No clinical signs or strong variations in weight were detected during the entire experiment. Following euthanasia on day 28 post-infection, brain tissues and eyes were collected from all mice and the parasite load was quantified by RT-qPCR. Results are presented in Fig. 7 and Table 3. In terms of parasite DNA detection in brain tissues, 6 out of 8 animals tested PCR positive in the placebo-control group, and in the BKI-1748 treatment group *Toxoplasma* DNA could be detected in 5 out 8 animals. All animals were PCR positive in the artemiside treated group, and 7 out of 8 tested positive in the group that had received the drug combination. When comparing cerebral parasite loads, treatment with BKI-1748 and combined artemiside-BKI-1748 resulted in a significant reduction of parasite numbers in the brain compared to the placebo-treated infection control while treatment with artemiside alone did not have an impact on cerebral parasite burden (Fig. 7A). Analysis of the IgG responses by ELISA revealed that there were no significant differences in IgG levels among the different groups (data not shown). However, closer analysis showed that 2 out of 8 mice each in the placebo control group and in the BKI-1748 group, and 1 out of 8 mice in the combined treatment group, had remained seronegative. Overall, combining artemiside with BKI-1748 had no effect when compared to BKI-1748 treatment alone, thus under the conditions used in this experiment, the promising *in vitro* results could not be translated to the *in vivo* situation.

## 4. Discussion

The development of a drug to reduce the impact of toxoplasmosis is a challenging task. The parasite has a complex life cycle consisting of stages that exhibit different metabolic requirements, and the potential emergence of drug resistance poses a potential problem (Alday and Doggett, 2017; Morrison and Tomley, 2016). One more recently developed compound for the treatment of toxoplasmosis is BKI-1748. Previous *in vitro* studies have shown that the CDPK1 inhibitor BKI-1748 was efficient in limiting *T. gondii* proliferation *in vitro*, and in *T. gondii* oocyst-infected mice, BKI-1748 treatment led to reduced transplacental transmission to newborns and as a consequence, increased pup survival. However, loss of offspring was not entirely prevented (Imhof et al., 2021).

One approach to increase efficacy and tackle drug resistance is the development of combination therapies, which have been developed for numerous infectious diseases, including those caused by parasites (Andrews et al., 2014). For instance, the classical treatment for acute toxoplasmosis is a pyrimethamine-sulfadiazine combination, which targets two independent steps of the folic acid synthesis pathway leading to a lack of purines. Leucovorin is added as a supplement to prevent folic acid deprivation in the host. In case of adverse effects associated with sulfa-drugs, pyrimethamine-clindamycin combination treatment can be applied, exerting a dual action with clindamycin inhibiting translation in the apicoplast (Hernandez et al., 2017). To combat malaria caused by *P. falciparum*, artemisinin-based combination therapy (ACT) is employed, whereby currently artemisinin-derivatives such as dihydroartemisinin, artesunate and artemether are applied in combination with lumefantrine, mefloquine, amodiaquine, sulfadoxine/pyrimethamine, piperaquine and chlorproguanil/dapsone (Rosenthal, 2003; Kappagoda et al., 2011). Combining compounds with different modes of action or various targets can lead to synergistic effects, that might be exploited to reduce toxicity problems or lower the risk of resistance formation (Sun et al., 2016). Another candidate drug that could be incorporated into ACT is the amino-artemisinin artemiside, which is rapidly metabolized into artemisox and then artemisone (Gibhard et al., 2021).

In order to test our initial hypothesis that the amino-artemisinin derivative artemisone and of the CDPK1 inhibitor BKI-1748 may have synergistic effects, we assessed the *in vitro* activities of either individual or combined drug, against *T. gondii* tachyzoites *in vitro* in a short-term assay. In this assay, BKI-1748 had a lower IC_50_ compared to artemisone (46 versus 92 nM, respectively). These values fit well to previously reported data (Müller et al., 2023; Imhof et al., 2021; Dunay et al., 2009). In addition, both compounds exhibited almost identical IC_90_ values (269 and 271 nM). Combining BKI-1748 and artemisone at equimolar concentrations did not result in increased efficacy in terms of IC_50_ but halved the IC_90_. This is in good agreement with our initial hypothesis that both compounds may act synergistically when combined. In contrast, HFF were not affected by artemisone concentrations above 2 μM (Müller et al., 2023) and BKI-1748 concentrations of 10–40 μM had not impact on HFF, HepG2or CRL-8155 cells (Anghel et al., 2020).

This synergism does, however, not result in a complete clearance of tachyzoites *in vitro*. Host cells infected with a high number of tachyzoites and treated post infection with artemisone harbored parasitophorous vacuoles containing numerous tachyzoites during the treatment, as shown by immunofluorescence. Combined BKI-1748 and artemisone treatment resulted in parasitophorous vacuoles of diminished size, like what has been observed with a BKI-ELQ combination (Anghel et al., 2021). The determination of the minimal inhibitory concentration of artemisone alone and in combination is difficult. A previous study (Müller et al., 2023) employing long-term *in vitro* treatments with artemisone up to 20 days has shown that *T. gondii* ME-49 tachyzoite proliferation was suppressed for few days, but parasites rapidly adapted to the presence of artemisone, leading to the generation of a resistant strain that still underwent proliferation at 2.5 μM. Thus, the inhibition mediated by artemisone is, at best, of transient nature.

Synergistic effects of BKI-1748 and artemisone were also apparent upon ultrastructural investigations of host cell layers treated post infection. As shown earlier (Müller et al., 2023) parasites treated with artemisone located within a parasitophorous exhibited an intact nucleus and secretory organelles, but lacked structurally intact mitochondria, implying that the mitochondrion is one of the drug targets. In addition, the cytoplasm was extensively vacuolized, indicative for metabolic impairment. In contrast, BKI-treated parasites had formed MNCs, like BKI-treated *N. caninum* (Ojo et al., 2014; Imhof et al., 2021), and formed a large intracellular mass composed of newly formed zoites with intact secretory organelles, largely intact mitochondria, and no or only very few cytoplasmic vacuoles. Newly formed nuclei were not randomly distributed within these complexes but concentrated in one region, while most apical parts of newly formed zoites were located at the opposite end of the MNC. Similar multinucleated stages were described upon *in vitro* treatment of *T. gondii* with the pyrazolo (2,3-*d*)pyrimidine compound BKI-1294 (Winzer et al., 2015) and with an unrelated drug, diclazuril (Lindsay et al., 1995). Multinucleated forms were also reported upon BKI-treatment of the related apicomplexans *N. caninum, Sarcocystis neurona* and *Besnoitia besnoiti* (Ojo et al., 2014, 2016; Jiménez-Meléndez et al., 2017). MNCs obtained through treatment of *N. caninum* with BKI-1294 have been proposed to be designated as baryzoites (Greek barys = massive, bulky, heavy, inert) as they represent an enlarged parasitic stage that ensures survival of these parasites at increased drug concentrations during prolonged periods of time (Winzer et al., 2020b).

Upon artemisone-BKI-1748 combination treatment, small MNCs enclosed within a parasitophorous vacuole displaying discernible apicomplexan hallmarks including apical complex, rhoptries, and structurally intact mitochondria were still visible after 48 h. However, at later timepoints these MNCs were heavily distorted and most likely not viable anymore. Many parasites were single tachyzoites or tachyzoite residues, situated within a parasitophorous vacuole surrounded by a seemingly still intact parasitophorous vacuole membrane, but the cytoplasmic organization appeared heavily distorted, and in many cases parasites had accumulated irregularly shaped intracytoplasmic electron dense bodies. Most likely, these electron dense bodies are not dense granules, as these would be round and not irregularly shaped, but could potentially represent accumulated acidocalcisomes. Acidocalcisomes are found in diverse organisms, including bacteria and human cells, but also apicomplexan parasites. They possess an acidic matrix that contains several cations including calcium, magnesium, sodium, potassium, zinc, and iron bound to short and long polyphosphate chains. Their matrix is acidified through the action of proton pumps such as a vacuolar proton ATPase and a vacuolar proton pyrophosphatase. Their functional roles are attributed to ion storage, pH homeostasis, and osmoregulation, and Ca^2+^ release of the acidocalcisomes in trypanosomes has been implicated in cellular bioenergetics and cell death (Ramakrishnan et al., 2018). We speculate that *T. gondii* might upregulate those organelles as a reaction to the drug-induced stress (Attias et al., 2020), but this aspect needs to be further investigated. Although it is possible that the observed drug-induced features, especially at the later timepoints, are just representative of dying parasites and not mirror specific drug-induced changes, it appears conceivable that combining BKI-1748 treatment with artemisone imposes increased structural damage and increased toxicity upon *T. gondii.* In addition, no regrowth of *T. gondii* tachyzoites was observed after 12 days of *in vitro* treatment with the BKI-1748-artemisone combination, both at 1 μM (data not shown).

Based on these *in vitro* findings, we embarked on *in vivo* studies employing a pregnant *T. gondii* ShSp1 occyst infection model (Imhof et al., 2021) The safety of BKI-1748 upon oral application in pregnant and non-pregnant *T. gondii* infected mice has been demonstrated earlier (Imhof et al., 2021; Anghel et al., 2020). To investigate safety of artemisone during pregnancy, artemiside, which is rapidly metabolized to artemisox and then artemisone (Gibhard et al., 2021; Watson et al., 2021) was applied to non-infected pregnant BALB/c mice at 20 mg/kg/day during 5 days starting at day 9 post-mating (Anghel et al., 2020). This resulted in impaired fertility and/or abortion of all pups, while no adverse clinical signs were noted in the adult animals. Studies carried out in various other animal models have also demonstrated that artemisinin derivatives can cause foetal death and congenital malformations including defective embryonic erythropoiesis and vasculogenesis/angiogenesis during early embryogenesis. However, clinical data on the safety profile artemisinin-derivatives in pregnant women suggest that there is no risk of miscarriage, stillbirth, or congenital malformation, nor low birth weight, associated with these drugs (D’Alessandro et al., 2020). As a consequence, *in vivo* efficacy studies were limited to non-pregnant mice infected with *T. gondii* oocysts.

Infection with *T. gondii* oocysts did not result in clinical signs in any of the mice. Oral application of BKI-1748, artemiside and the combination of the two drugs did also not cause any adverse effects and did not reduce the total numbers of positive brains, but in terms of cerebral parasite load, significant differences were noted. All brains in the artemiside treated group and 6 out of 8 brains in the control group were PCR positive, and there was no overall difference in cerebral parasite loads in those two groups as assessed by real time PCR. The two animals in the control group that were PCR-negative also tested negative by ELISA, and the lack of antibodies indicates that they were not properly infected, which mirrors a potential technical issue during gavage.

In contrast, BKI-1748 and the combined BKI-1748-artemiside treatment both had a profound impact on cerebral infection since the cerebral parasite load was significantly reduced in those two groups compared to the control group. Thus, while artemiside treatment did not prevent parasite dissemination to the brain, it did also not impair the activity of BKI-1748. However, it is evident that by applying a combination of both drugs, the excellent efficacy of BKI-1748 is not improved further.

The fact that artemiside is not active *in vivo* could be related to the pharmacokinetic properties of the compound. It was reported earlier that upon oral application of artemiside at 50 mg/kg to male C57BL6 mice, maximal plasma levels of artemisone were in the range of 2 μM after 1 h, and then declining to 0.2 μM after 3 h. In our study, treatments were carried out at 20 mg/kg, thus is likely that the treatment failure is due to insufficient exposure in the circulation. The route of drug delivery most likely also makes a difference. When assessed earlier for efficacy against acute infection with tachyzoites of the type II *T. gondii* PRU-Luc-GFP strain applied intraperioneally in outbred CD1 mice (Dunay et al., 2009), artemiside was applied subcutaneously at 10 mg/kg/day for 8 days, resulting in a 60% survival rate. To evaluate effects on the reactivated toxoplasmosis, inbred gamma interferon IFN-ko C57BL6 mice susceptible to both the acute and reactivation phases were challenged with tissue cysts of the same strain followed by treatment with sulfadiazine to suppress acute infection and allow development of tissue cysts. After sulfadiazine treatment was stopped, conversion of bradyzoites to tachyzoites occurred, and also in this case treatment with artemiside (10 mg/kg/d) for 8 days prolonged survival in 80% of the animals, while the control mice died within 10 days. However, artemiside was not able to eradicate the chronic infection and likely does not act directly on bradyzoites (Dunay et al., 2009). In the light of these finding one could envisage a combination treatment in which BKI-1748 is applied perorally, but artemiside could be administered by subcutaneous injection.

In any case, while adjustments to the treatment protocol, such as the timing of treatment start, extension of treatment duration or alterations in the dosage and formulations, could potentially improve efficacy, it is evident that the encouraging results obtained *in vitro* could not be translated to the murine model under the conditions used in this study. A potential way to improve *in vivo* efficacy could be the formulation of drugs such as solid dispersions, in nano/microparticles, polymeric micelles, nanosuspensions, lipid-based nanocarriers, and other formulations which have been developed for other compounds to increase solubility and improve oral bioavailability (e.g. Du et al., 2022). Another approach would be to use a synthetic endoperoxide, with similar mechanisms of action to artemisinins, that have long plasma exposures, such as OZ439 under investigation for malaria (Dong et al., 2017).

In conclusion, this study shows that it is difficult reproduce promising *in vitro* results in suitable *in vivo* models. Therefore, care must be taken not to extrapolate the efficacy of test compounds from mere *in vitro* findings to the situation in animal or human patients, which are, again, different from rodent models.

## Figures and Tables

**Table 1.** Inhibition constants (IC_50_, IC_90_ = drug concentrations inhibiting proliferation by 50% and 90%, respectively) of artemisone against *T. gondii* RH beta-galactosidase-expressing tachyzoites grown in HFF monolayers as calculated from the data depicted in Fig. 1. IC values were calculated using the logit-log algorithm and are indicated with 95% confidence intervals. The Hill factor H corresponds to the absolute value of the slope of logit = f (log concentration).

Compound	IC_50_	H	IC_90_

BKI-1748	46 [37–49] nM	1.54	269 [216–286] nM
Artemisone	92 [73–115] nM	3.05	271 [215–339] nM
BKI-1748+Artemisone	42 [38–47] nM	2.74	138 [125–154] nM

**Table 2:** Pregnancy interference assay in non-infected pregnant BALB/c mice (n = 6 per group) using BKI-1748 and artemiside, both emulsified in corn oil and applied from day 7–11 of pregnancy at 20 mg/kg/day.

Groups	Number of pregnant mice	Mice per group	Number of dead pups within the first 2 days	Total number of pups (litter size)	Neonatal mortality	Postnatal mortality

BKI-1748*	4	6	2	20	0/20	0/20
Artemiside	2	6	abortion, no pups found	abortion, no pups found	-	-
Corn oil	4	6	1	23	0/23	1/23

* data from Imhof et al. (2021).

**Table 3:** Cerebral *T. gondii* infection in mice of control and drug treated groups as assessed by PCR. The numbers of positive brains were not significantly different (Chi-Square test for independence; p > 0.02).

Groups	*T. gondii* positive brains

Artemiside	8/8
BKI-1748	5/8
BKI-1748 + Artemiside	7/8
Placebo control	6/8

## Data Availability

Data will be made available on request.
